# The effect of visual and proprioceptive feedback on sensorimotor rhythms during BCI training

**DOI:** 10.1371/journal.pone.0264354

**Published:** 2022-02-23

**Authors:** Hanna-Leena Halme, Lauri Parkkonen

**Affiliations:** 1 Department of Neuroscience and Biomedical Engineering, Aalto University School of Science, Espoo, Finland; 2 MEG Core, Aalto Neuroimaging, Aalto University School of Science, Espoo, Finland; Cook Children\’s Health Care System: Cook Children’s Medical Center, UNITED STATES

## Abstract

Brain–computer interfaces (BCI) can be designed with several feedback modalities. To promote appropriate brain plasticity in therapeutic applications, the feedback should guide the user to elicit the desired brain activity and preferably be similar to the imagined action. In this study, we employed magnetoencephalography (MEG) to measure neurophysiological changes in healthy subjects performing motor imagery (MI) -based BCI training with two different feedback modalities. The MI-BCI task used in this study lasted 40–60 min and involved imagery of right- or left-hand movements. 8 subjects performed the task with visual and 14 subjects with proprioceptive feedback. We analysed power changes across the session at multiple frequencies in the range of 4–40 Hz with a generalized linear model to find those frequencies at which the power increased significantly during training. In addition, the power increase was analysed for each gradiometer, separately for alpha (8–13 Hz), beta (14–30 Hz) and gamma (30–40 Hz) bands, to find channels showing significant linear power increase over the session. These analyses were applied during three different conditions: rest, preparation, and MI. Visual feedback enhanced the amplitude of mainly high beta and gamma bands (24–40 Hz) in all conditions in occipital and left temporal channels. During proprioceptive feedback, in contrast, power increased mainly in alpha and beta bands. The alpha-band enhancement was found in multiple parietal, occipital, and temporal channels in all conditions, whereas the beta-band increase occurred during rest and preparation mainly in the parieto-occipital region and during MI in the parietal channels above hand motor regions. Our results show that BCI training with proprioceptive feedback increases the power of sensorimotor rhythms in the motor cortex, whereas visual feedback causes mainly a gamma-band increase in the visual cortex. MI-BCIs should involve proprioceptive feedback to facilitate plasticity in the motor cortex.

## 1. Introduction

Brain–computer interfaces (BCI) have been developed rapidly over the recent years, and especially their clinical application in neurorehabilitation has gained interest [1]. BCI training has been suggested as an alternative or complement to traditional physical therapy in cerebral stroke rehabilitation [2]. Many clinical studies have reported improved function of a paretic upper limb after BCI use [3], but the long-term effects remain debatable [4].

A BCI, by definition, consists of three parts: the user’s brain, the computer which processes user’s brain signals and controls stimuli, and the interfaces between these two. The measurement interface is typically electroencephalography (EEG) or magnetoencephalography (MEG), and the feedback interface is visual, auditory, somatosensory, or proprioceptive stimulation delivered to the user in real time. The purpose of the feedback is to guide the user to elicit the desired brain activity. When designing a BCI, it is crucial to select the feedback modality according to the targeted neural function. While BCI control can be achieved using any feedback, not all feedback modalities necessarily alter the user’s brain activity as intended [5, 6]. Especially in the case of rehabilitation, the BCI should give feedback that promotes appropriate brain plasticity and helps patients regain their impaired functionality, e.g. upper limb movements. This typically means that the desired brain activity is rewarded with positive feedback, which might involve e.g. proprioceptive stimulation of the impaired limb.

Motor imagery (MI), i.e. mental imagery of movement, can be used for controlling a BCI. The feedback signal of current MI-BCIs can be delivered through various modalities ranging from simple visual feedback to robotic orthoses and virtual reality [1]. However, it has been reported that in healthy subjects, proprioceptive and haptic feedback in MI-BCI facilitates motor-cortex activation more efficiently than visual feedback [7, 8]. In addition, feedback consistent with the imagined movement might improve the decoding accuracy in online signal classification [9].

In patient studies, the effect of different BCI feedback modalities remain controversial. Improved upper limb functionality has been reported in several clinical BCI studies involving end-effector robots [10–14], functional electrical stimulation (FES) [15–18] and visual feedback [19–21]. However, a recent review article [6] evaluated the effect sizes of several clinical studies and found that FES was the only feedback modality which significantly improved upper limb function after MI-BCI training. The effect size in studies involving BCIs with proprioceptive and visual feedback was insignificant, and thus the impact of these feedback modalities on rehabilitation cannot be confirmed.

In general, practice improves motor skills in healthy subjects, and the improvement is often manifested as measurable neurophysiological changes. Short-term changes related to motor learning are e.g. increase of alpha rhythm over sensorimotor and occipital regions [22], decrease in movement-related beta rhythm suppression [23–26] and alpha rhythm suppression [27, 28] as well as increased beta power at rest [26, 29] in the sensorimotor cortex. In addition, higher prefrontal gamma-band activity has been related to better MI-BCI performance [30] and movement-related gamma power increase has been observed during motor learning [31]. It is thus likely that gamma-band power changes are also linked to motor and MI-BCI skill acquisition.

The neurophysiological effects of MI-BCI training in healthy participants are less clear than those of motor practice. Previous studies have reported increased SMR suppression in the contralateral hemisphere [32–34] and decreased connectivity of associative areas [34], as well as increased motor cortical excitability and beta-band connectivity changes [35]; however, these changes occurred over a time span of several days or weeks. Short-term BCI training has been found to increase the strength [36] and lateralization [37] of SMR suppression. However, in most previous studies the neurophysiological changes have been assessed between a few experimental runs, i.e. the signals have been averaged over each run and those averaged signals have been compared. Thus, it is still unclear whether short-term learning of MI-BCI skills causes linear SMR power or modulation change over a single measurement session.

In this study, we examine the neurophysiological changes in healthy subjects performing MI-BCI tasks with two different feedback modalities. One group performed the BCI task with visual and the other group with proprioceptive feedback. We assume that activity in the motor cortex is enhanced in the proprioceptive feedback group during training, since the given feedback is very similar to what the imagined movement would produce if executed. Furthermore, we hypothesize that similar functional changes are not elicited by purely visual feedback.

## 2. Materials and methods

### 2.1. Measurements

Two MEG datasets involving MI-BCI experiments were included in this study. In both cases, MEG was recorded at the MEG Core of Aalto University School of Science with a 306-channel Elekta Neuromag^™^ Vectorview system (MEGIN Oy, Helsinki, Finland) using a sampling frequency of 1 kHz and passband of 0.1–330 Hz. Five head-position indicator (HPI) coils were attached to the subject’s scalp for head position estimation and alignment with a standard coordinate system. Visual stimuli were delivered on a back-projection screen located 100–150 cm from the subject’s eyes via a projector outside the magnetically shielded room. Raw MEG data were continuously written in 300-ms segments to a network-transparent ring buffer [38, 39] hosted by the MEG acquisition workstation (6-core Intel Xeon CPU at 2.4 GHz, 64-bit CentOS Linux, version 5.3). The buffer was read over a local network connection by the stimulus computer (Intel Core i7-4771 CPU at 3.5 GHz, 64-bit Ubuntu Linux, version 12.04-LTS in Data 1 and version 14.04 in Data 2). This computer processed the MEG data in real time using functions implemented in the MNE-Python software [40], controlled the proprioceptive stimulators (in Data 2) and presented the visual stimuli using PsychoPy version 1.83 [41].

Both studies were approved by Aalto University research ethics committee. The research was carried out in accordance with the guidelines of the declaration of Helsinki, and all subjects gave written informed consent prior to the MEG measurements.

**Fig 1:**
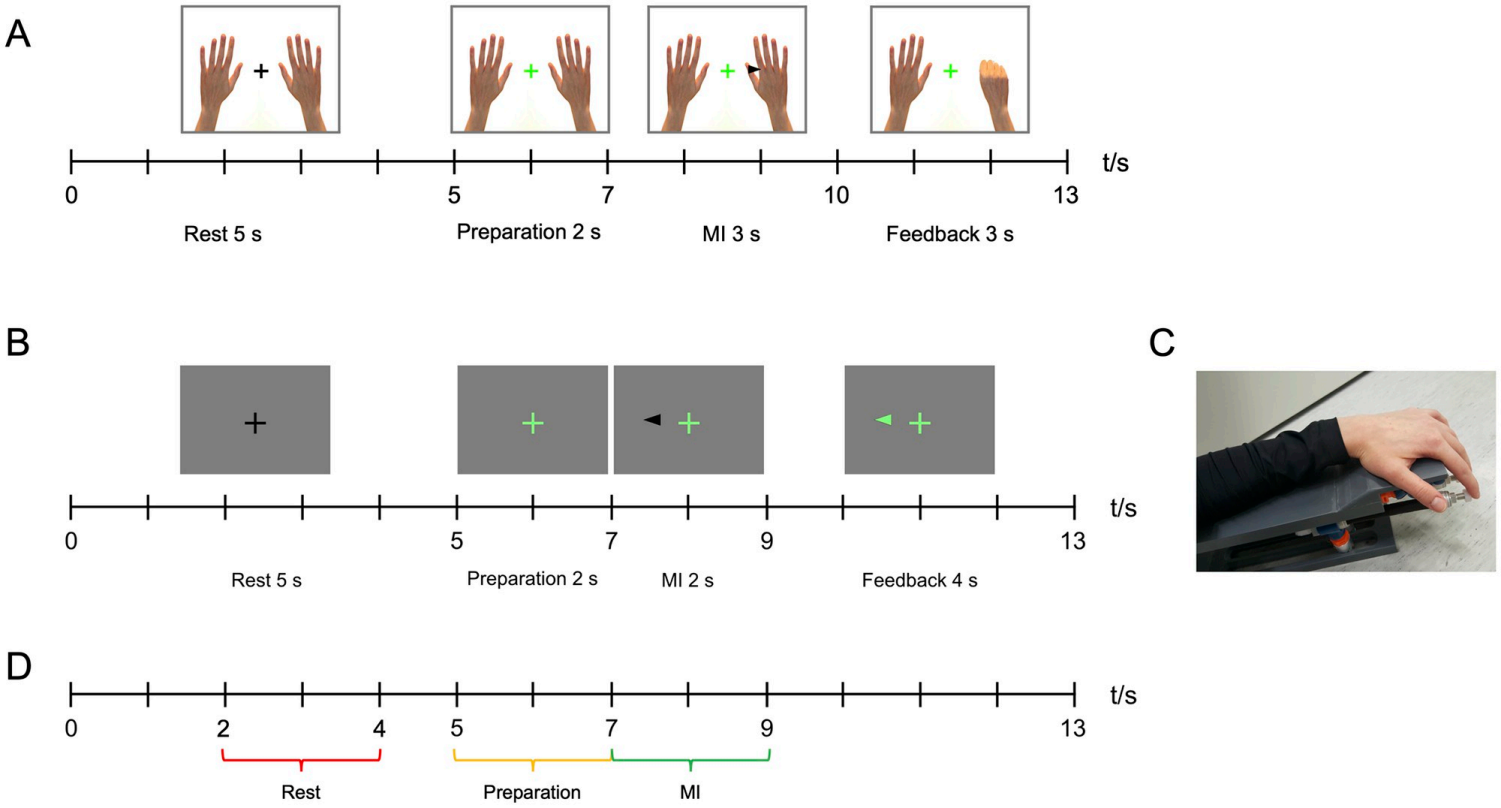
Stimulus paradigms. Stimulus paradigms in (A) Data 1 and (B) Data 2, and (C) the proprioceptive stimulator used in collecting Data 2. The time windows (D) used for data analyses.

The stimulus paradigms for Data 1 and Data 2 are shown in Fig 1A and 1B, respectively. The black arrow indicates which hand the subject should imagine moving. More detailed information about the stimuli and online classification for controlling the feedback can be found in [42] for Data 1. Data 2 were collected with a similar feedback paradigm as the data in our earlier study [43], although the online classifier was different.

### 2.2. Data 1: Visual feedback

These data were collected for a previous study [42] and included nine healthy volunteers (5 females, 4 males, aged 25.8 ± 1.4 years, all right-handed by self-report). None of the subjects reported any neurological illnesses or motor deficits, and all subjects had normal or corrected-to-normal vision. None of the subjects had previous experience in BCI training. One subject was discarded from further analyses because no reliable online feedback was given due to technical problems in the MEG measurement. Thus, 8 subjects were included in the final dataset.

During each trial, the subjects performed either left- or right-hand MI, followed by visual feedback in the form of an animation of one or both hands opening and closing (i.e., grasping) based on the measured MEG signals. The subjects were instructed to imagine sequential finger-tapping movements, and it was emphasized that they should perform kinesthetic instead of visual imagery. Each subject practiced the task by performing overt finger-tapping for a few minutes before the MEG measurement. The measurement comprised 40 left-hand and 40 right-hand MI trials, presented in a random order and split into two runs.

For the online classification, power spectral densities in the range 8–30 Hz were calculated for 48 parietal gradiometers with a multitaper method as implemented in MNE-Python, with 7 discrete prolate spheroidal sequence (DPSS) windows and a bandwidth of 1 Hz. 50 most relevant PSD features, according to chi-squared statistics, were selected and classified with linear discriminant analysis (LDA). The online binary classification result determined the feedback such that the probability for each hand was linearly converted to the degree of hand grasp on each side, involving 20 degrees ranging from a fully open hand to a fully closed fist. A trial was considered successful if the probability of the target hand was higher than that of the opposite hand.

### 2.3. Data 2: Proprioceptive feedback

These data are previously unpublished. The dataset included MEG measurements from 16 healthy volunteers (9 males, 7 females, aged 30.4±6.6 years, all right-handed by self-report). None of the subjects reported any neurological illness or motor deficits, and all subjects had normal or corrected-to-normal vision. Subjects having any metal objects inside their body or other MEG contraindications were excluded. Three subjects had participated in our previous BCI experiment [42] and are also included in Data 1. The others had no previous BCI experience. Two subjects were discarded from further analyses, one due to excessive artifacts in the MEG signal, and the other because only two MI-BCI runs were measured due to technical problems. Thus, 14 subjects were included in the final dataset.

During each trial, the subjects performed either left- or right-hand MI, followed by proprioceptive feedback. The subjects were instructed to imagine movement similar to that delivered by the proprioceptive stimulators (see details below), which was demonstrated in an initial run involving passive movements with the stimulators. Also in this study, the participants were specifically told to perform kinesthetic imagery. The measurement comprised 75 left-hand and 75 right-hand MI trials, presented in a random order and split into three runs. For a reliable comparison between the two datasets, only 80 first trials of Data 2 are included in further analyses. 40 first trials for both left-hand and right-hand MI are included in left- and right-hand MI specific analyses.

The proprioceptive feedback was delivered by 8 elastic pneumatic artificial muscles (PAM; DMSP-10-100 AM-CM, diameter 10 mm, length of the contracting part 100 mm; Festo AG & Co, Esslingen, Germany) attached to fingers 2–5 of both hands with tape and embedded in custom-made plastic frames. During the experiment, the subject’s hands and arms were resting on the plastic frames and the PAMs were touching the fingertips horizontally from beneath the frame (see Fig 1C). The PAMs were connected to the frame via movable hinges, so that the stimulator system could be adjusted to the subject’s hand. When the stimulator was triggered, pressurized air was conveyed to the PAMs, making them contract and thus flex the subject’s fingers towards the palm. Each PAM contracted for 500 ms and then returned to the resting length, extending subject’s fingers back to the initial position. Thus, in case of a successful MI trial, the stimulators moved the subject’s fingers in a sequential manner for 2 s in total.

### 2.4. Online analysis

The MEG signals were classified online using a multi-task learning paradigm in which a classifier was trained with 18 healthy subjects’ MEG data; see our previous publication for details [43]. The MI trials were baseline corrected (baseline –1…0 s), filtered to 8–30 Hz and decimated by 10 to speed up the computations. For subjects 1–4, the classification was done using the whole trial. For the rest of the subjects, the classification was done in three overlapping 2-s windows (–1…1 s, –0.5…1.5 s and 0…2 s). A weight vector **W** was obtained from the training data, initially optimized for a set of 64 parietal gradiometers and a time window of –1…1 s from the cue. For each window, the signals of all measurement channels were concatenated to form a feature vector of size [1, *n*_timepoints * *n*_channels]. The sign of the dot product of the feature vector and weight vector **W** determined the class of the current window: < 0 left; > 0 right. The final classification of the trial was defined as follows:

- If the first window was classified as the target hand, the trial was considered correctly classified.
- If the first window was not classified as the target hand, the sum of the classifications for all three windows was used to determine the class of the trial in the same way as for a single window.

For the above classification scheme, the chance level was 62.5%. It was considered that a positively biased classifier is more encouraging for the naïve subjects.

### 2.5. Offline preprocessing

The signal space separation method [44], as implemented in the MaxFilter software (version 2.2.22; MEGIN Oy, Helsinki, Finland), was applied to the data to suppress external magnetic interference, align the head positions to the standard position and orientation and interpolate bad channels. Only signals from gradiometers were retained for further analyses. The raw signals were divided into discrete trials, band-pass filtered to 3–45 Hz and decimated to 250-Hz sampling frequency. Artifacts related to cardiac and muscular activity, eye movements, power line interference and other high-amplitude noise components were removed with independent component analysis (ICA) and the automatic artifact detection algorithm implemented in MNE-Python [40].

### 2.6. Offline analysis

We estimated the spectral power of MEG signal using the multitaper method, as implemented in MNE-Python, with 7 DPSS windows and a bandwidth of 0.5 Hz. The power was estimated for each trial in 3 separate time windows: *rest* (–5.0 … –3.0 s before beginning of MI), *preparation* (–2.0 … 0.0 s), and *MI* (0.0 … 2.0 s). The spectral power was calculated for frequencies 4–40 Hz and separately for each gradiometer.

The power values were first averaged over all subjects and gradiometers in order to get a grand-average power for each trial. These average power values were plotted and a trendline was calculated for each plot using a linear least-squares regression; for this we used the *polyfit* function implemented in Matlab (R2018b; Mathworks Inc., MA, USA). Correlation between trial index and spectral power was estimated using Matlab function *corrcoef* in each frequency range and condition. Thus, we could roughly estimate whether the power in alpha-, beta- and gamma-band was increasing or decreasing over the measurement session.

For a more detailed analysis, the power values were subjected to a generalized linear model (GLM). For this analysis, we used the *glmfit* function implemented in Matlab. The GLM design matrix contained trial index and subject age as explanatory variables. For simplicity, we assumed the power change over trials would be linear, and thus decided to use the linearly increasing trial index as an explanatory variable. The power values were log-transformed prior to GLM analysis to meet the normality assumption. First, the power values were averaged over all gradiometers and the model was fitted for each frequency in the range 4–40 Hz. Similar analysis was done using only the signals from parietal gradiometers. After that, we calculated the average power for alpha (8–13 Hz), beta (14–30 Hz) and gamma (30–40 Hz) bands at each gradiometer and fitted the model separately for all gradiometers. The aim of the mentioned analyses was to find 1) the frequencies showing significant linear increase over trials, and 2) the channels in which alpha, beta or gamma power showed significant linear increase over trials, i.e. the effect of explanatory variable *trial* in the generalized linear model was statistically significant (*p* < 0.05).

Next, we calculated the suppression percentage of frequencies 4–40 Hz in parietal channels during MI, relative to rest. The suppression values were also subjected to the GLM analysis to find out whether the MI-related modulation of these frequencies changed during BCI training. In addition, we estimated the subject-specific alpha and beta frequencies by finding the most prominent peaks in the signal spectrum averaged over parietal gradiometers. The individual frequency ranges were defined as peak ±2 Hz and peak ±4 Hz for alpha and beta bands, respectively. Thereafter, the average alpha and beta suppression in parietal channels was calculated for each subject at these frequency ranges, and the GLM model was fitted to the average suppression values. Finally, GLM was fitted for suppression values at each parietal channel, separately for alpha and beta as well as right-hand and left-hand MI. These analyses were restricted to the parietal region since it was assumed that MI-related modulation of sensorimotor rhythms (SMR) should occur in this region.

In both frequency- and channel-specific GLM analyses, the resulting *p*-values were corrected for multiple comparisons with the Benjamini–Hochberg false discovery rate method [45] implemented in Matlab R2018b.

## 3. Results

### 3.1. Online classification accuracy

The online classification accuracies are reported in Tables 1 and 2 for Data 1 and Data 2, respectively.

**Table 1:** Online classification accuracies (percent) when delivering visual feedback (Data 1).

Subject	Run 1	Run 2	Mean
1	49	59	**54.0**
2	68	61	**64.5**
4	49	56	**52.5**
5	93	83	**88.0**
6	51	78	**64.5**
7	51	42	**46.5**
8	71	63	**67.0**
9	59	66	**62.5**
**Mean**	**61.4**	**63.5**	**62.4**

**Table 2:** Online classification accuracies (percent) when delivering proprioceptive feedback (Data 2).

Subject	Run 1	Run 2	Run 3	Mean
1	68	62	54	**61.3**
2	70	70	74	**71.3**
3	54	78	64	**65.3**
4	58	58	64	**60.0**
5	84	92	78	**84.7**
6	70	64	66	**66.7**
7	88	78	74	**80.0**
8	72	78	80	**76.7**
10	68	60	64	**64.0**
11	76	80	72	**76.0**
12	78	70	64	**70.7**
13	86	90	80	**85.3**
14	84	84	82	**83.3**
15	84	62	70	**72.0**
16	80	84	86	**83.3**
**Mean**	**74.7**	**74.0**	**71.5**	**73.4**

### 3.2. Power of alpha, beta, and gamma oscillations

**Fig 2:**
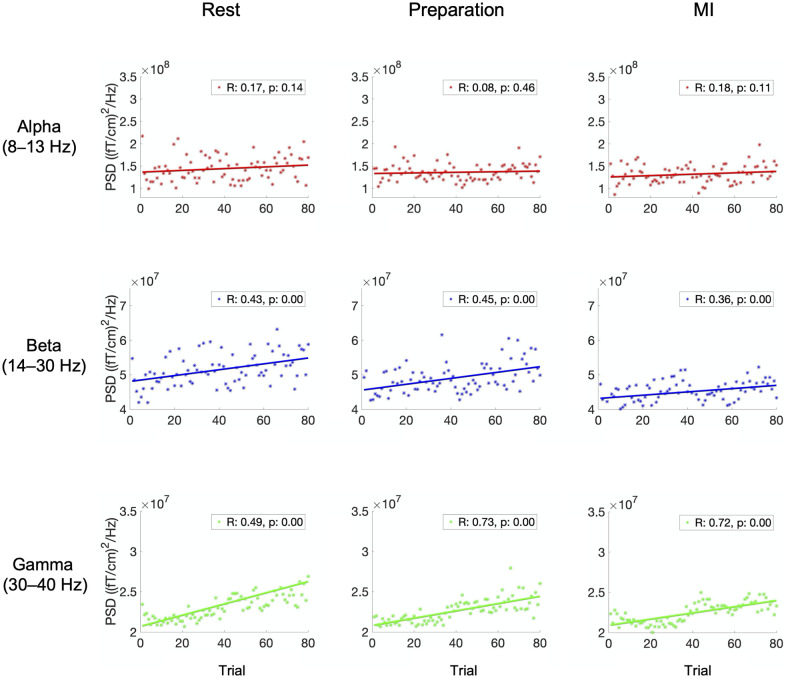
Alpha, beta, and gamma power over trials in Data 1. Power of alpha-, beta-, and gamma-band oscillations, averaged over all subjects and gradiometers, as a function of trial index when delivering visual feedback (Data 1). Correlation coefficient *R* and the corresponding *p*-value are shown in each plot.

Fig 2 shows the alpha-, beta- and gamma-band power, averaged over all subjects and channels, in each trial of Data 1 (visual feedback) during rest, preparation and MI. Correlation coefficients *R* and the corresponding *p*-values are shown for each plot. The correlation coefficients and trendlines show that alpha power did not increase over trials. However, beta and gamma power showed a linear increase during rest, preparation, and MI.

**Fig 3:**
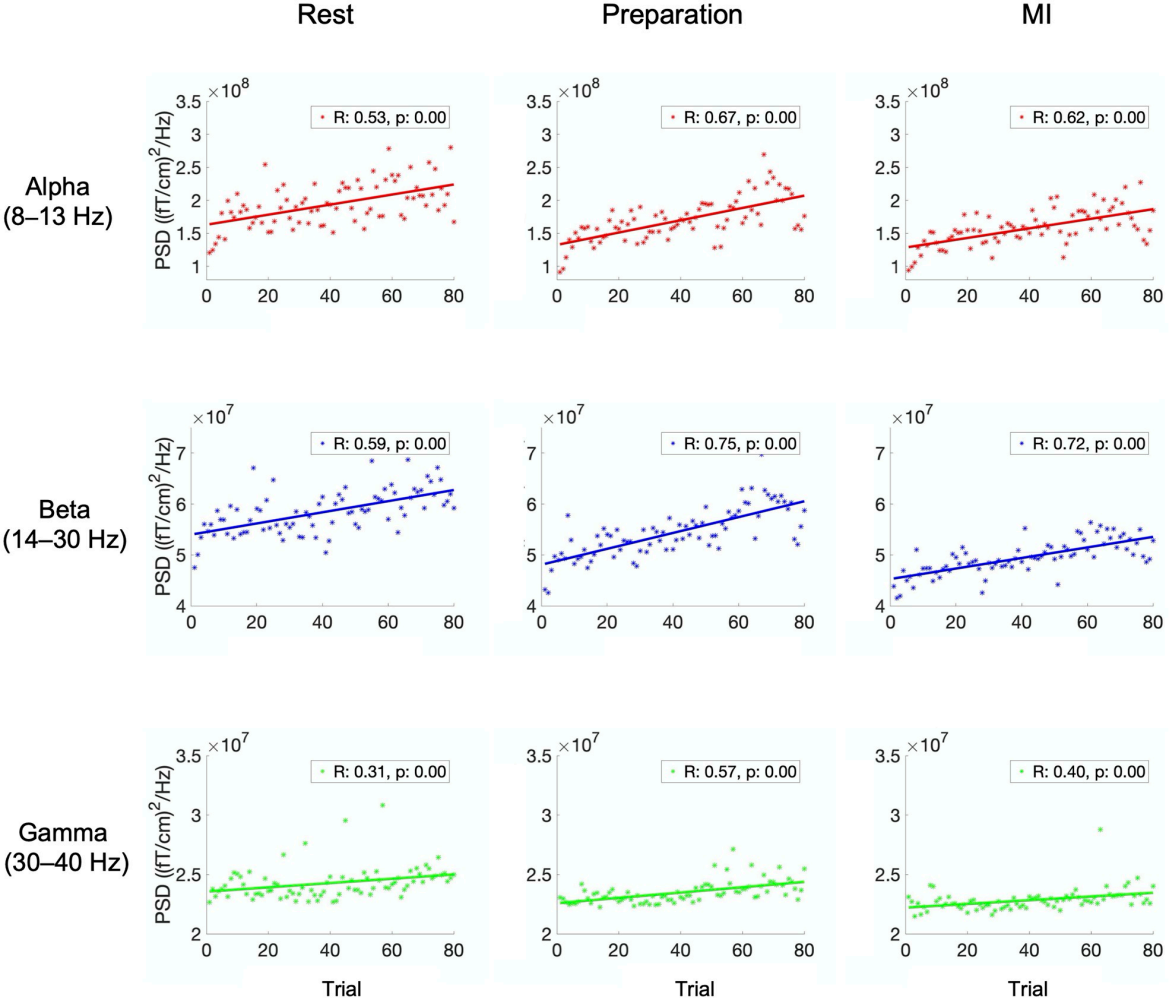
Alpha, beta, and gamma power over trials in Data 2. Power of alpha-, beta-, and gamma-band oscillations, averaged over all subjects and gradiometers, as a function of trial index when delivering proprioceptive feedback (Data 2). Correlation coefficient *R* and the corresponding *p*-value are shown in each plot.

Similarly, Fig 3 shows the power values for alpha-, beta- and gamma-band, averaged over all subjects and channels, in each trial of Data 2 (proprioceptive feedback) during rest, preparation and MI. Correlation coefficients and the corresponding *p*-values are shown for each plot. The correlation coefficients and trendlines indicate a linear increase over trials for alpha-, beta- and gamma-band power during rest, preparation, and MI.

### 3.3. Frequency-specific analyses

**Fig 4:**
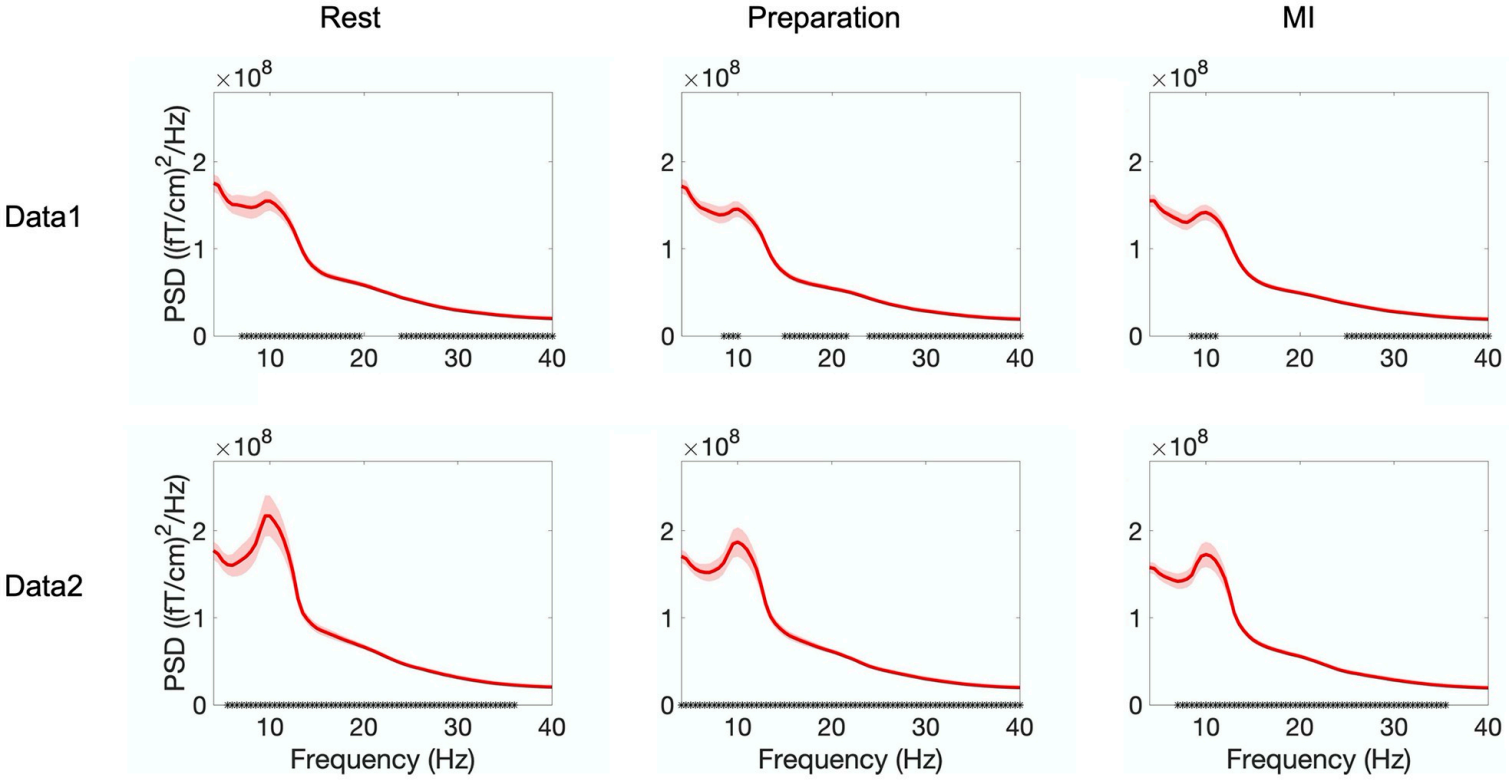
Frequency spectra averaged over all gradiometers. Frequency spectra (average across all gradiometers) when delivering visual (Data 1) and proprioceptive (Data 2) feedback, separately during rest, preparation and MI. Frequencies showing a significant (*p* < 0.05) linear power increase over trials are marked on the frequency axis with asterisks. Shaded areas represent the standard error of the mean (SEM) over subjects.

Fig 4 shows the frequency spectra during rest, preparation and MI for Data 1 and Data 2. The plots show an average over all gradiometers, all trials, and all subjects. In Data 1 (visual feedback), a significant linear power increase over trials was observed in 7–20 Hz and 24–40 Hz for rest; in 8–10 Hz, 15–22 Hz and 24–40 Hz for preparation; and in 8–11 Hz and 25–40 Hz for MI. In Data 2 (proprioceptive feedback), the power increased over trials in frequency range 5–36 Hz in rest, 4–40 Hz in preparation and 7–36 Hz in MI.

**Fig 5:**
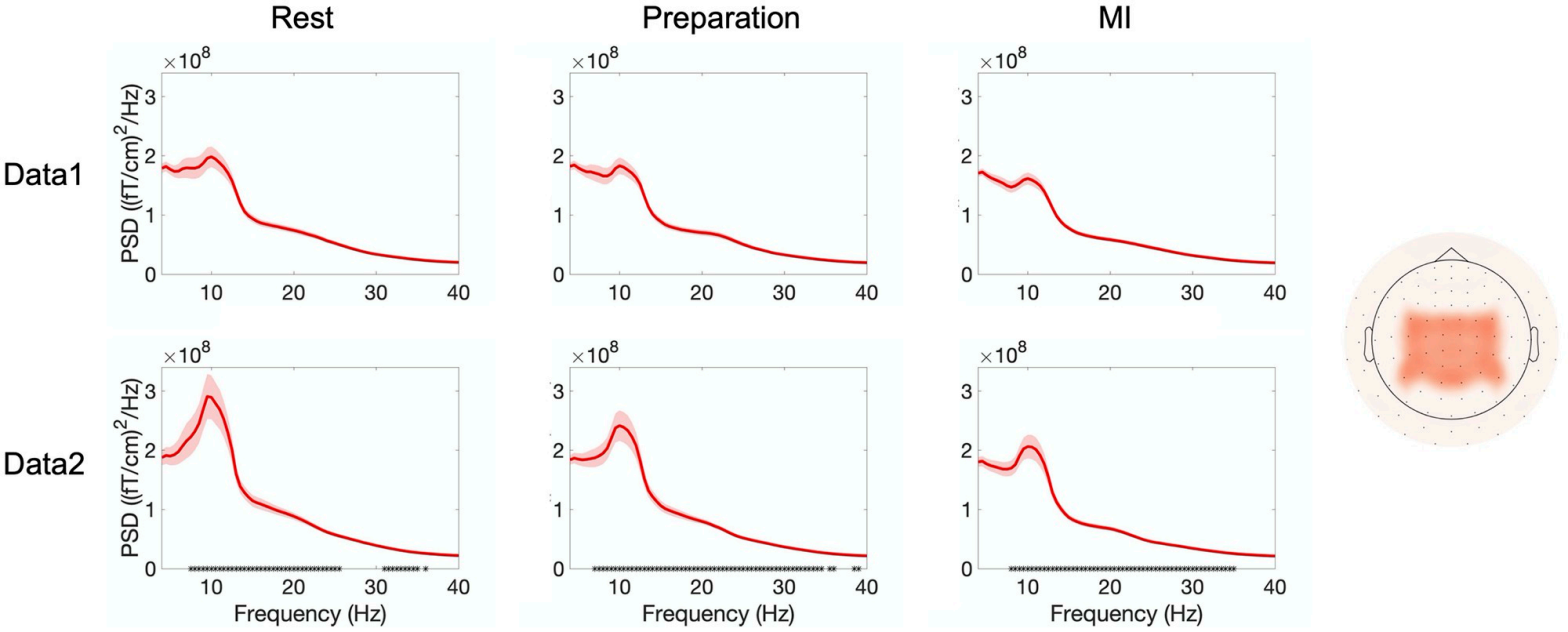
Frequency spectra averaged over parietal gradiometers. Frequency spectra (average across parietal gradiometers, shown in the rightmost column) when delivering visual (Data 1) and proprioceptive (Data 2) feedback, separately during rest, preparation and MI. Frequencies showing a significant (*p* < 0.05) linear power increase over trials are marked on the frequency axis with asterisks. Shaded areas represent the standard error of the mean (SEM) over subjects.

Fig 5 shows the frequency spectra and suppression during rest, preparation and MI for Data 1 and Data 2. The plots show an average over parietal gradiometers (channels included in the analyses shown in the rightmost column), all trials and all subjects. In Data 1 (visual feedback), no significant power increase was found in any frequency 4–40 Hz in any condition. In contrast, in Data 2 (proprioceptive feedback), power increased significantly in 7–26 and 31–35 Hz for rest; 5–35, 36 and 39 Hz for preparation; and 8–35 Hz for MI.

### 3.4. Channel-specific analyses

**Fig 6:**
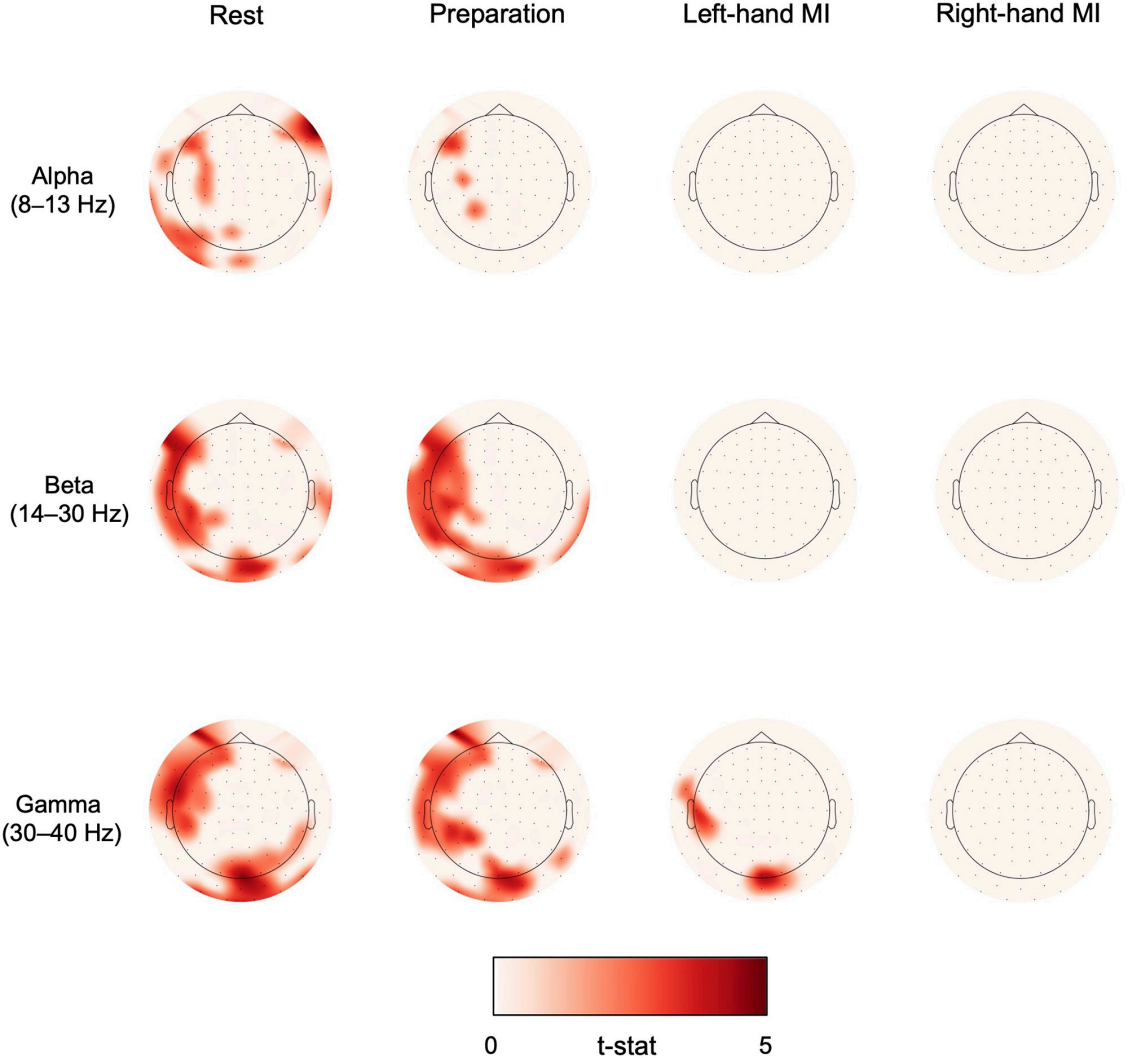
Channel-specific power increase in Data 1. MEG gradiometers in which a linear power increase over trials was statistically significant (*t*-statistic shown for channels in which *p* < 0.05) for alpha-, beta- and gamma-band oscillations when delivering visual feedback (Data 1).

**Fig 7:**
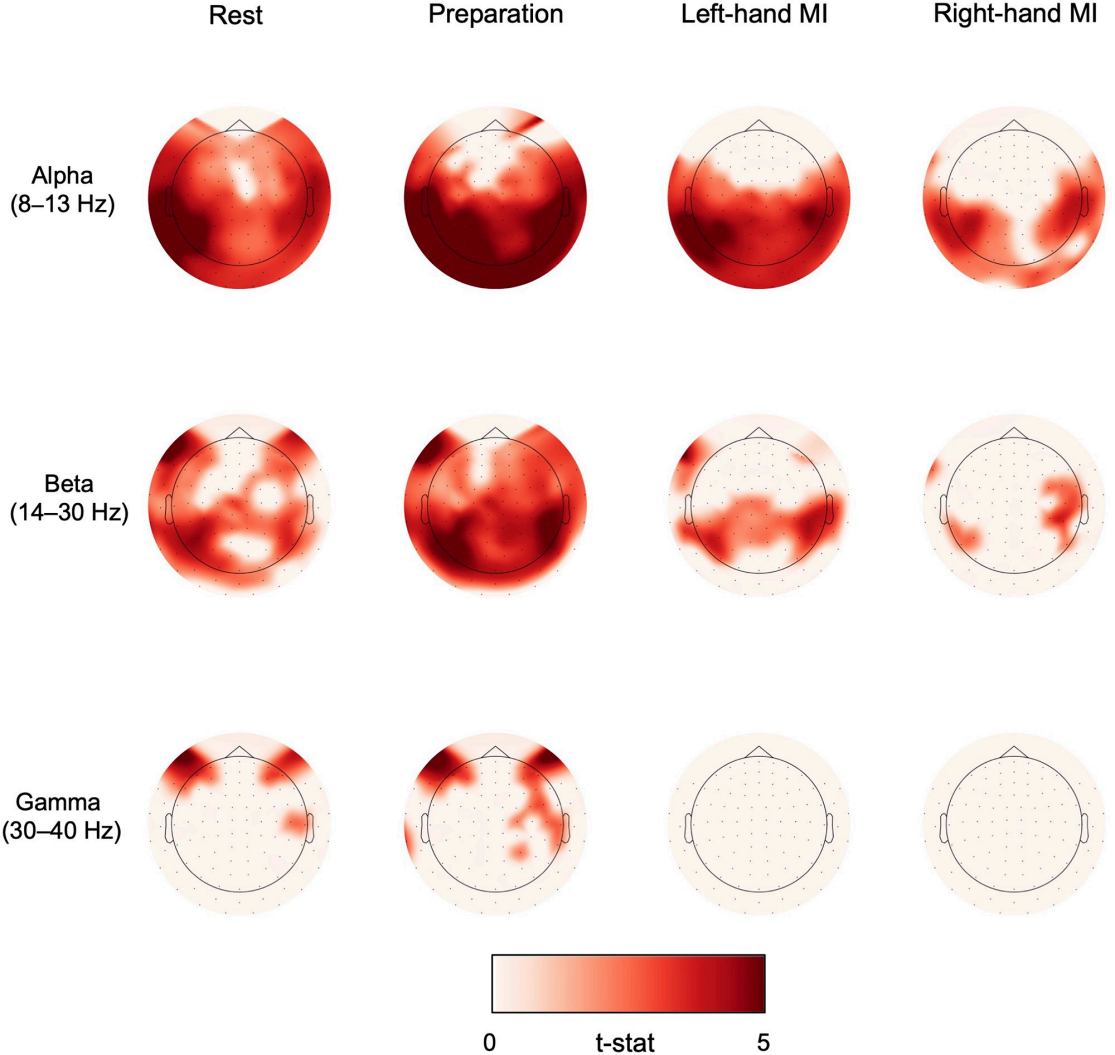
Channel-specific power increase in Data 2. MEG gradiometers in which a linear power increase over trials was statistically significant (*t*-statistic shown for channels in which *p* < 0.05) for alpha-, beta- and gamma-band oscillations when delivering proprioceptive feedback (Data 2).

Channels in which alpha-, beta- or gamma-band power increased linearly (*p* < 0.05) over trials during rest, preparation and MI are shown in Fig 6 for Data 1 and in Fig 7 for Data 2.

In Data 1 (visual feedback), alpha power increased significantly during rest at left frontal, parietal, and occipital channels, and during preparation at left frontal and parietal channels. Beta power increased as well during rest and preparation in occipital and left fronto–parietal channels. For right-hand MI, no power increase was found in any channel in either alpha, beta or gamma frequencies. For left-hand MI, there was a significant gamma-power increase extending from occipital to bilateral temporal and frontal channels.

In Data 2 (proprioceptive feedback), alpha power increased significantly in all four conditions. At rest, the increase was seen in almost all channels. During preparation and MI, power increase was significant in bilateral parietal and occipital channels, roughly corresponding to motor and visual cortices. The effect was stronger for the left- than right-hand MI. Beta power increase was found during rest in frontal, parietal, and occipital regions and during preparation in nearly all channels. During MI, the significant power increase was focused bilaterally in the parieto–temporal region close to the motor cortex. Again, the power increase was more pronounced during left-hand than right-hand MI.

Gamma-band power increased in a few channels in the right parietal region during rest and preparation. Gamma-band increase was also found in bilateral frontal regions in these conditions. However, as these frontal channels are close to the eyes it is likely that this finding is simply an artefact caused by small eye movements during the task. We investigated this issue further by calculating the spatial topographies of high gamma (40–100 Hz) frequencies during the first two experimental runs, as eye-movement -related spectral content should be present in those high frequencies as well. In addition, time–frequency plots for the high-gamma range, averaged over frontal gradiometers, were created for the first two runs. The results are shown in S1 Fig. The spatial topographies show clear localization of high-gamma frequencies to the sensors close to both eyes. The power during rest and preparation seems to increase at those channels from Run 1 to Run 2 while this is not the case for MI. The time–frequency plots showed increased high-gamma spectral power during rest and preparation, compared to baseline (–5…–4 s). Especially during preparation, the high-gamma modulation increased from Run 1 to Run 2. We cannot thus rule out that ocular artifacts explain the gamma-band increase in frontal channels in Data 2.

### 3.5. Change in SMR suppression

Suppression of frequencies 4–40 Hz during MI, compared to rest, did not show a significant change in any frequency in either Data 1 (visual feedback) or Data 2 (proprioceptive feedback) in the course of the experiment. In both Data 1 and Data 2, the strongest suppression was found in the beta range, i.e. 15–25 Hz. Alpha (8–12 Hz) rhythm was suppressed during MI in Data 2 but not in Data 1; in fact, in Data 1, the power of alpha seems to increase during MI relative to rest.

**Fig 8:**
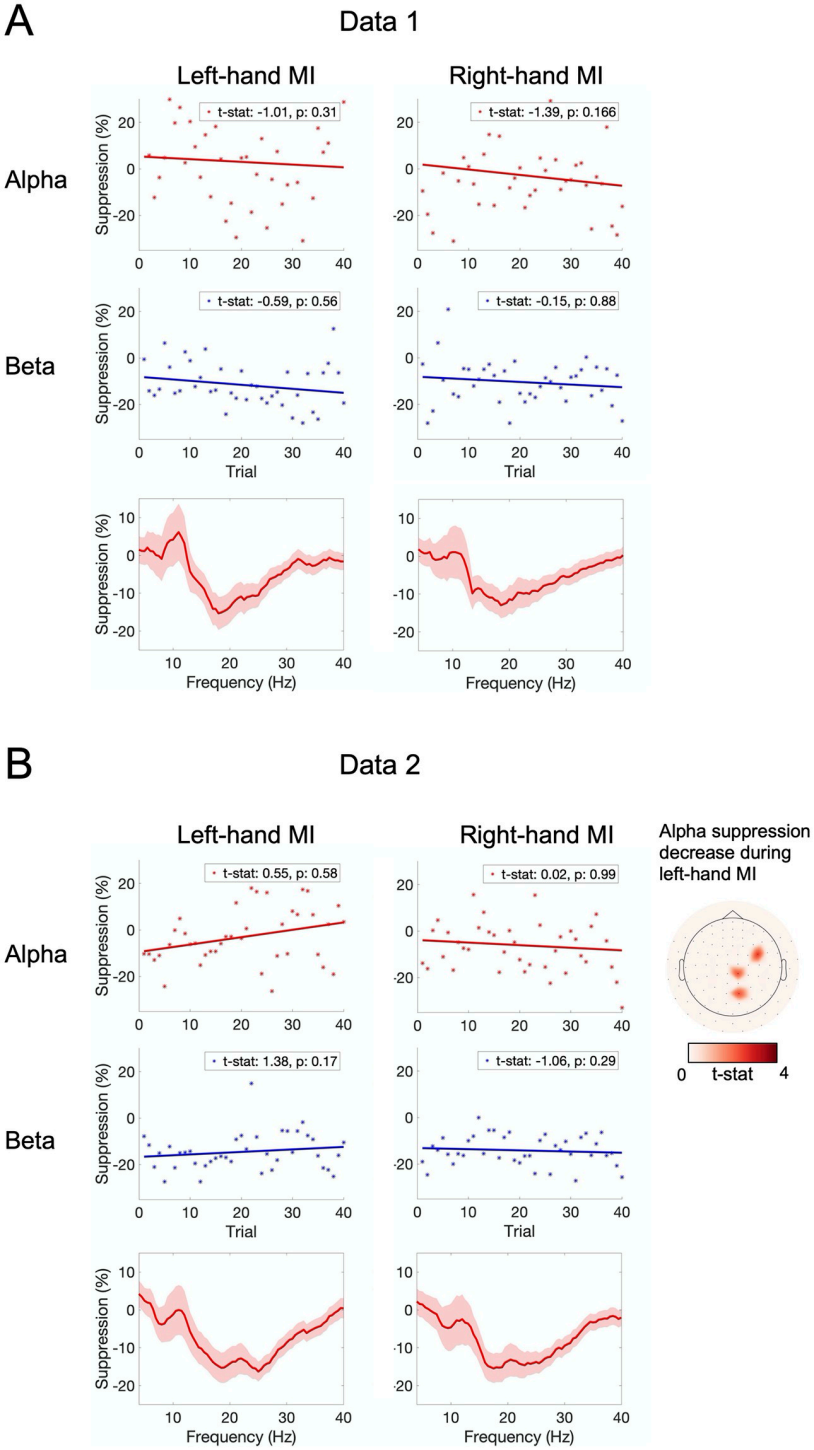
Suppression of alpha and beta frequencies during MI. Alpha and beta suppression for each trial for left- and right-hand MI, and suppression at each frequency in range 4–40 Hz, in (A) Data 1 (visual feedback), (B) Data 2 (proprioceptive feedback). The solid lines show linear regression. *T*-statistics and *p*-values obtained from GLM are shown in each plot. In the frequency-specific suppression plots, shaded areas represent the standard error of the mean (SEM) over subjects. For Data 2, the channels in which alpha suppression decreased over training are shown in the rightmost column. All analyses are done using only the data from parietal gradiometers.

GLM analysis did not reveal any significant suppression changes in the signals averaged over parietal gradiometers in either Data 1 or Data 2. When fitted to signals of individual channels, GLM showed a statistically significant decrease of alpha suppression during left-hand MI for Data 2 at three contralateral channels, i.e. the suppression got weaker at these channels during training. Other significant changes were not found for alpha or beta suppression for either dataset. The results are summarized in Fig 8.

## 4. Discussion

In the current study, we evaluated neurophysiological changes related to MI-BCI training with two different feedback modalities. We found that proprioceptive feedback corresponding to the imagined hand movement elicited a significant linear increase in alpha- and beta-band power over the short training session. The alpha-band power increase was prominent in widespread brain regions during rest, preparation, and MI, whereas the beta-band increase was mainly observed in bilateral parieto–occipital regions during rest and preparation and bilateral parietal regions during MI.

The results are consistent with studies stating that motor imagery activates the sensorimotor cortices [46, 47] and concurrent proprioceptive feedback enhances this effect by providing stimuli to the peripheral nerves, thus closing the sensorimotor loop [9]. In the current study, the power increase with proprioceptive feedback (Data 2) in the alpha and beta ranges, i.e. in the SMR range, might be related to increased cortical inhibition during training. After some training, the MI-BCI task requires less mental effort, which is reflected in the increased alpha power over motor and visual regions. This finding is in line with the theory stating that alpha-band oscillations inhibit the function of task-irrelevant brain areas [48, 49]. Another possibility is that increased alpha power reflects the improved synchronization of neural activity within the associated brain regions, possibly related to working memory processes [50, 51].

Increase of beta power in Data 2 during rest and preparation could be related to learning of the BCI task and concurrent increased prediction of an expected stimulus [52]. Increased resting-state beta power related to motor training has been observed in previous studies [26, 29], suggesting a practice-induced effect. Moreover, sensorimotor beta-band activity has been associated with maintenance of the current motor state [53], e.g. keeping still while waiting for movement instructions. With regards to this hypothesis, our results could suggest that the subjects learned to remain mentally idle during rest and preparation, which resulted in increased beta power in sensorimotor areas during those intervals.

In addition, beta power during MI increased in sensors close to the hand motor regions bilaterally. This finding can be interpreted such that the MI-related beta-band suppression in the motor cortex decreased or became more localized to contralateral hand motor area over training, because the subjects learned to perform MI more effortlessly. Similar results have been obtained previously in motor learning tasks [23, 25, 54] and during BCI use [55]. Such an effect was not observed during MI-BCI training with purely visual feedback in the current study. In fact, no alpha or beta power changes were found in the sensorimotor region when visual feedback was used in BCI training.

Visual feedback resulted in increased alpha (~10 Hz) and high beta/gamma-band (25–40 Hz) power during MI. Our analyses revealed that the power enhancement occurred mainly in the occipital and left temporal regions, i.e. not in the motor cortex. Gamma-band coherence in task-engaging brain regions is found to play a role in associative learning [56], and thus the increasing gamma power over trials when using visual feedback (Data 1) might be related to learning of BCI use. Although power increase was observed also in left parietal channels during rest and preparation, it was the most pronounced in occipital channels, which suggests that the subjects were probably relying more on visual than kinesthetic imagery when performing MI. Therefore, the functional changes were observed mainly on the visual cortical regions. It was shown previously that visual and kinesthetic imagery activate different brain regions [57], and the increase of visual cortical activity during BCI training is in line with this finding. Even though the subjects were instructed to perform kinesthetic imagery, the purely visual feedback in Data 1 and especially the lack of somatosensory feedback has probably encouraged visual imagery of hand movements instead.

The changes in SMR suppression over trials were only observed for proprioceptive feedback (Data 2) during left-hand MI in the alpha band; the suppression of alpha diminished over training in three contralateral channels. Otherwise, no effect of BCI training to SMR suppression was found in either dataset. The results might be explained by the short training time. Previous studies have reported enhanced SMR modulation over long-term MI-BCI training lasting several days or weeks [32–34, 58]. A recent study reported enhanced SMR modulation after BCI training compared to that measured before training [36]; however, it is unclear whether the enhancement of modulation was linear over the BCI experiment as the trial-to-trial change was not assessed with linear regression. Regarding the high inter-trial and inter-subject variation in SMR modulation, it is possible that significant changes can only be seen when comparing the average modulation levels of multiple experimental runs. Also, considering that MI-induced SMR modulation is inherently much weaker than that caused by overt movements [47], it is not very surprising that possible subtle training-induced effects do not reach statistical significance during a single measurement session. Thus, it remains to be investigated whether longer training time significantly affects the SMR modulation.

Although we could not find significant linear increase or decrease in SMR modulation, we found certain differences in SMR suppression between the datasets. In case of visual feedback (Data 1), alpha rhythm was not suppressed during MI, whereas in case of proprioceptive feedback (Data 2) both alpha and beta rhythm were clearly suppressed. As the suppression was analyzed only over the parietal channels (i.e. over the motor cortex), this finding implies that proprioceptive feedback is able to modulate both the alpha and beta components of the SMR, while visual feedback only modulates the beta-band oscillation.

With proprioceptive feedback (Data 2), significant gamma-band power increase was observed during rest and preparation in bilateral frontal channels close to both eyes. This finding is most likely due to small eye movements, since the larger movements were eliminated in preprocessing with ICA. Miniature saccades which are not time-locked to external stimuli have been reported to induce transient power increase in range 20–100 Hz in EEG [59], which can be confused with neuronal gamma-band activity. Similar phenomenon has been reported in MEG [60]. These findings are in line with our results, as also beta-band (14–30 Hz) power showed an increase in channels near the eyes during rest and preparation. In addition, a similar effect could be seen to a lesser extent in Data 1. We also analyzed the time–frequency plots and spatial topographies of high-gamma frequencies for Data 2 and found that the 40–100 Hz power was the most prominent at the same locations as the gamma-band increase. It thus seems that the occurrence of saccades increased during BCI training, perhaps because the subjects got more tired during the experiment and could no longer continuously focus on the fixation cross. The grey background and simple high-contrast visual stimuli in Data 2 were probably more tiring for the subjects’ eyes than the photo-realistic hand images in Data 1, which explains why the saccade artifact was not that prominent in Data 1. The design of visual stimuli should be carefully considered in future BCI experiments to avoid such artifacts.

Although in clinical BCI studies, functional electric stimulation (FES) has been the most effective feedback modality [6], proprioceptive stimulators might be worth more investigation in the context of neurorehabilitation. In many previous studies [10, 11, 13, 14, 61], feedback has been given by end-effector robots, in which the movement range is typically limited to e.g., hand opening and closing. In addition, FES typically stimulates one muscle or muscle group at a time, causing rather gross movements of the upper limb. However, more precise movements might be needed for the improvement of fine hand motor functions. The pneumatic stimulator used in the current study can be configured to deliver various hand and finger movement sequences according to each BCI user’s needs. Additionally, the proprioceptive stimulators are comfortable and easy to attach, and thus more pleasant for patients.

This study has some limitations. First, a direct statistical comparison between the two feedback groups was not done, as the groups involved partially the same subjects and were not therefore completely independent, but not suitable for pairwise comparison either. Thus, we decided to omit between-group analyses in this study, especially since the essential conclusions can be drawn also from the single-group analyses. Second, the training time in both experiments was very short (less than an hour), and more significant neurophysiological changes may occur over longer training periods. However, it is noteworthy that we already observed significant neurophysiological changes during these short training sessions. Third, in Data 1, the visual feedback was graded according to the classification probabilities, and in Data 2 the proprioceptive feedback was either given or not given. This essential difference between the feedback paradigms might have also affected the subjects’ learning, although in both paradigms it was clear for the subject whether the MI trial was successful or not. Also, in both studies the feedback was delivered a few seconds after the beginning of MI, because the online classifiers were not designed for continuous decoding. While this is certainly an issue that needs to be addressed in future studies, it does not affect the comparison of feedback modalities in this study, since the feedback delay was similar in the two experiments. Last, although our assumption of a linear power change over trials seems to be valid in these data, we acknowledge that this is just a hypothesis and non-linear changes, especially during long-term MI-BCI learning, are also possible.

Together, our results imply that a BCI with proprioceptive feedback can alter motor cortex function in healthy subjects. Power increase over trials was observed in SMR range and close to hand motor regions during MI. On the contrary, visual feedback did not elicit significant changes either in the SMR range or in the motor regions during MI. These results are consistent with our initial hypothesis that feedback similar to the imagined movement results in functional changes in the motor cortex, and this effect is not achieved with purely visual feedback. Thus, we could suggest that in neurorehabilitation of the motor cortex one should prefer proprioceptive over visual feedback or use a combination of both modalities.

## 5. Conclusions

Motor imagery combined with proprioceptive feedback increased the power of sensorimotor rhythms in healthy participants linearly over a short training session. No SMR power increase was observed when a similar BCI task was performed with visual feedback, indicating that visual stimuli alone are not able to change the motor cortex function during BCI training. Proprioceptive feedback in MI-BCIs can thus be suggested for further clinical experiments.

## Supporting information

S1 Fig High-gamma oscillations during proprioceptive stimulation. (A) Spatial topography of high-gamma (40–100 Hz) frequencies during rest, preparation and MI in the first two experimental runs of Data 2. (B) Time-frequency plots for frequencies 40–100 Hz, averaged over frontal gradiometers, in the first two runs of Data 2.
(PDF)
