# Supplementary material for: The effect of visual and proprioceptive feedback on sensorimotor rhythms during BCI training
Source: PLoS One. 2022 Feb 23;17(2):e0264354. doi: 10.1371/journal.pone.0264354 (PMC8865669; doi:10.1371/journal.pone.0264354)
Supplement: S1 Fig — (A) Spatial topography of high-gamma (40–100 Hz) frequencies during rest, preparation and MI in the first two experimental runs of Data 2. (B) Time-frequency plots for frequencies 40–100 Hz, averaged over frontal gradiometers, in the first two runs of Data 2. (PDF) [file pone.0264354.s001.pdf]

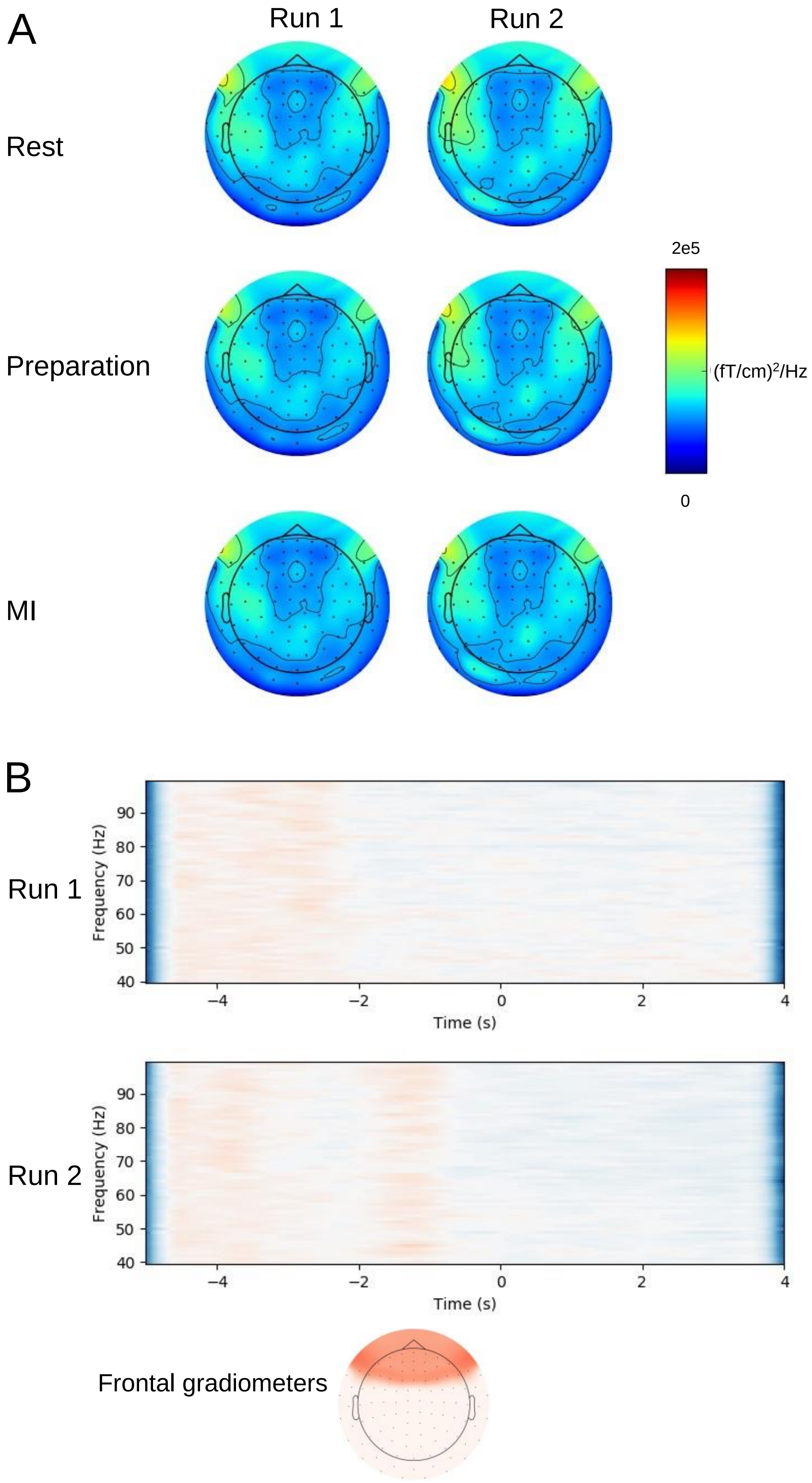

S1 Fig. High-gamma oscillations during proprioceptive stimulation. (A) Spatial topography of high-gamma (40–100 Hz) frequencies during rest, preparation and MI in the first two experimental runs of Data 2. (B) Time-frequency plots for frequencies 40–100 Hz, averaged over frontal gradiometers, in the first two runs of Data 2.
